# Rutin promotes osteogenic differentiation of mesenchymal stem cells (MSCs) by increasing ECM deposition and inhibiting p53 expression

**DOI:** 10.18632/aging.205546

**Published:** 2024-02-12

**Authors:** Dongyang Li, Wanru Yin, Chao Xu, Yongmin Feng, Xin Huang, Junfeng Hao, Chao Zhu

**Affiliations:** 1 Department of Rheumatology and Immunology, The Third Affiliated Hospital of Naval Medical University, Shanghai 201805, China; 2 Department of Science and Education, Jinqiu Hospital of Liaoning Province, Shenyang, Liaoning 110016, China; 3 Department of Dermatology, Shenyang Medical University, Shenyang 110034, China; 4 Department of Digestive Ward, Shenyang Red Cross Society Hospital China, Shenyang 110013, China; 5 Department of Nephrology, and Guangdong Provincial Key Laboratory of Autophagy and Major Chronic Non-Communicable Diseases, Affiliated Hospital of Guangdong Medical University, Zhanjiang 524001, China; 6 Department of General Practice Medicine, Shengjing Hospital of China Medical University, Shenyang 110022, China; 7 Department of Nephrology, The First Affiliated Hospital of China Medical University, Shenyang 110000, China

**Keywords:** MSCs, rutin, p53, ECM, osteogenesis

## Abstract

Mesenchymal stem cells (MSCs) are an important source of cells for bone regeneration. Although the utilization of MSCs along with growth factors and scaffolds is a beneficial clinical approach for bone tissue engineering, there is need for improvement on the effectiveness of MSC osteogenesis and differentiation. Rutin is a natural flavonoid and a major component for cell proliferation and bone development. However, studies on the mechanism through which rutin regulates osteogenesis and MSC differentiation are limited. Therefore, this study aimed to investigate the effect and mechanisms of rutin on osteogenic differentiation of MSCs. MSCs were extracted from umbilical cords and treated with rutin, followed by the examination of osteogenesis-related markers. Rutin treatment promoted the differentiation of MSCs towards the osteogenic lineage rather than the adipogenic lineage and increased the expression of osteogenic markers. RNA sequencing and bioinformatic analysis indicated that rutin regulated p53, a key gene in regulating the osteogenic differentiation of MSCs. Additionally, cellular experiments showed that rutin-induced decrease in p53 expression increased the formation of extracellular matrix (ECM) by promoting p65 phosphorylation and caspase-3 cleavage. Conclusively, this study demonstrates the importance of rutin in osteogenesis and indicates that rutin possesses potential pharmaceutical application for bone regeneration and bone tissue engineering.

## INTRODUCTION

Bone healing is a complex, slow, and unsatisfactory process. Regenerative medicine has faced considerable challenge in the restoration of bone tissue following serious injuries. Recent research indicates that mesenchymal stem cells (MSCs) possess high growth capacity and distinct characteristics in laboratory settings, making them a suitable cell resource for bone tissue engineering [1–3]. Notably, increasing the capacity of MSCs to differentiate towards the osteogenic lineage is the core goal of the bone regeneration field. Several factors, such as growth factors, transcription factors, and epigenetic factors [4, 5], regulate the process of determining MSC lineage.

Osteoblast differentiation begins with the expression of runt-associated transcription factor 2 (Runx2) during skeletal maturation. Runx2 can initiate the activation and differentiation of osteoblast in bone marrow stromal stem cells and increase the expression of genes related to bone formation, making it a key marker of bone formation [6, 7]. Moreover, Runx2 enhances the production of associated proteins in the extracellular matrix (ECM), such as collagen type 1 (COL-I), osteocalcin (OCN), and bone sialoprotein (BSP) [8].

Importantly, the differentiation of MSCs towards osteogenic and lipogenic lineages (bone, fat cell, or cartilage) is influenced by various growth factors, proteins, and signaling pathways, including the Wnt/β-catenin and TGF-β/BMP pathways, hedgehog proteins, Notch, and parathyroid hormone (PTH). Notably, these pathways act through specific transcription factors (TFs) that determine the cell lineage, such as osteogenic β-catenin, Runx2, and Osx for bone formation, adipogenic PPARγ, c/EBPα, Znf423, and TLE3 for fat cell formation, and chondrogenic SOX9 for cartilage formation [9].

Bioflavonoids possess several biological activities [10] and thus promising alternative growth factors for modulating cellular biological processes *in vitro*. Rutin, a natural bioflavonoid widely found in plants [11], possesses antioxidant effects and can be used to treat cardiocerebrovascular diseases, tumors, and inflammation [12–14]. Rutin is highly bioavailable, inexpensive, and safe [15], making it a suitable material for therapeutic studies. Moreover, a previous study showed that rutin promotes cell proliferation and osteogenic differentiation, effectively preventing osteoporosis [16].

However, little is known about their potential impact on MSC differentiation. Therefore, this study aimed to investigate the effect and molecular mechanisms of rutin in MSC differentiation, especially MSC differentiation towards the osteogenic lineage. Specifically, we examined the effect of rutin treatment on the differentiation of MSCs into osteocytes and adipocytes. Additionally, we investigated the expression of markers associated with osteogenesis (Runx2 and OPN) and adipogenesis (Pparg and Fabp4) during MSC differentiation. Overall, it is anticipated that this study would provide insights on the importance of rutin in the process of osteogenesis.

## RESULTS

### Rutin promotes osteogenic differentiation of MSCs

**Figure 1:**
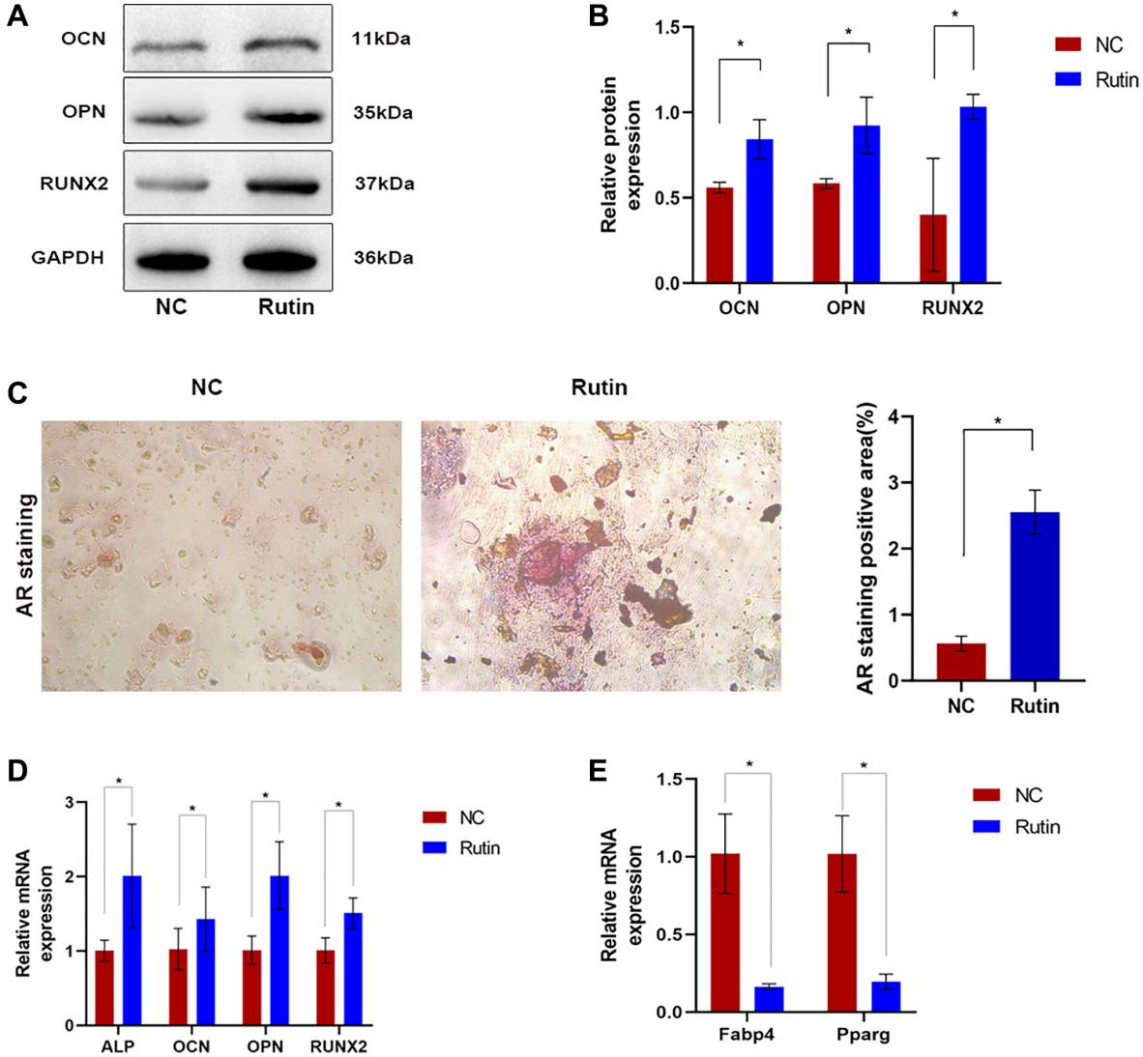
**The effect of rutin on MSC osteogenesis and differentiation.** (**A**, **B**) Western blot showed that rutin treatment in MSC culture improved the early and late osteogenic markers (OCN, OPN, RUNX2) level. (**C**) The AR staining. (**D**) Real-time RT-PCR analysis showed that the expression of early and late osteogenic markers (ALP, OCN, OPN, RUNX2) was significantly enhanced by rutin treatment. (**E**) Real-time RT-PCR analysis shows that the expression of adipogenic markers (Pparg, Fabp4) was significantly inhibited by treatment with rutin. The quantitative data were expressed as the mean ± S.D. of three independent experiments (^*^p < 0.05).

To determine the effect of rutin on osteoblastic differentiation, we examined the expression of osteogenesis-related genes in MSCs during osteogenesis induction. Compared with that in the control group, OCN, OPN, and Runx2 protein expression was significantly upregulated in rutin-treated cells (Figure 1A, 1B). Alizarin red (AR) staining showed that rutin treatment increased the number of calcified nodules in osteogenic MSCs (Figure 1C). Real-time quantitative reverse transcription PCR (qRT-PCR) showed that rutin treatment significantly upregulated the mRNA expression of ALP, OCN, OPN, and Runx2 compared with that in the control group (Figure 1D). MSCs maintain a delicate equilibrium in their differentiation potential into adipocytes and osteoblasts. Previous *in vitro* studies have shown that adipogenesis-promoting factors suppress osteogenesis, whereas osteogenesis-promoting factors inhibit adipogenesis [17]. Therefore, we investigated the effect of rutin on the adipogenic differentiation of MSCs. Compared with that in the control group, there was a significant decrease in the mRNA expression of adipogenesis-associated genes, including Pparg and Fabp4, in rutin-treated MSCs (Figure 1E). Overall, these results suggest that rutin may promote the osteogenic differentiation of MSCs.

### Transcriptome analysis reveals the mechanism of rutin in osteogenic differentiation of MSCs

**Figure 2:**
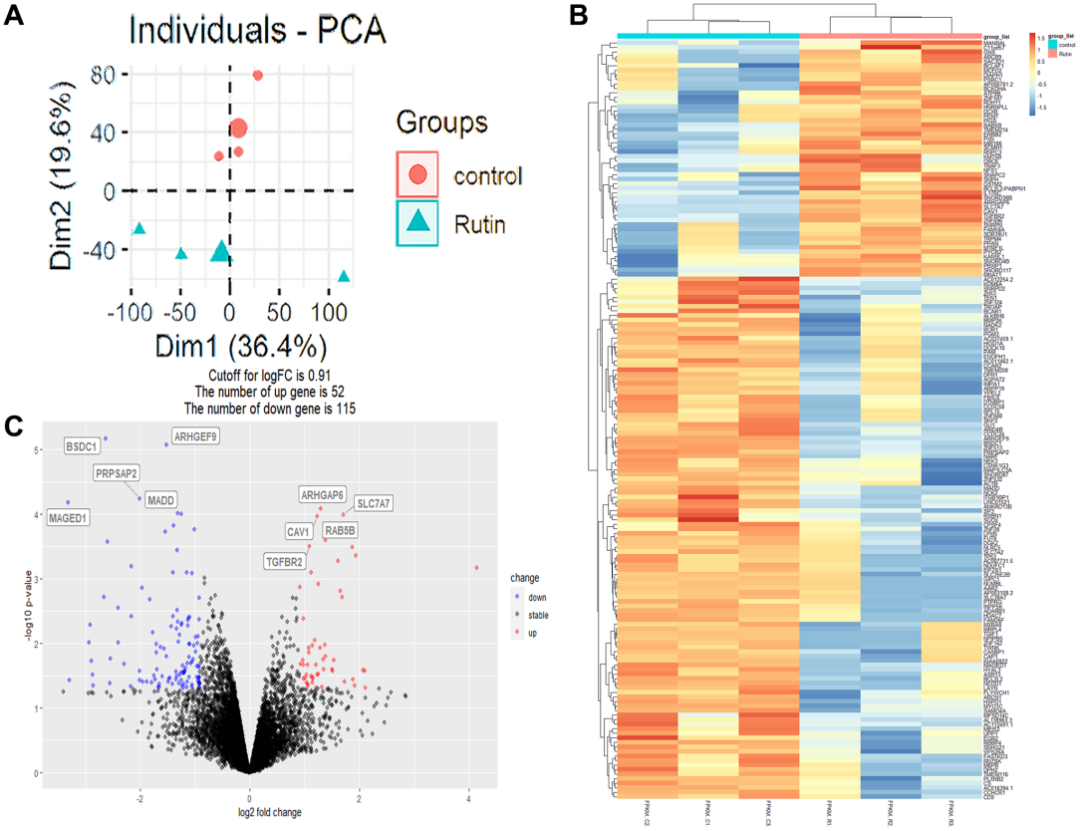
**Transcriptome analysis of rutin effects on osteogenic differentiation of MSCs.** (**A**) Principal component analysis (PCA) plot of the transcriptome data. (**B**) Heatmap of relative expression of genes across samples. (**C**) The volcano plot shows the differentially expressed genes (DEGs).

To investigate the regulatory mechanism by which rutin promotes the osteogenic differentiation of MSCs, we performed high-throughput sequencing of the transcriptomes of MSCs samples (rutin and control groups, *n* = 3). After processing and normalizing the raw sequencing data, principal component analysis (PCA) was performed on the samples. PCA showed that the samples formed two distinct clusters based on their characteristics (Figure 2A). Additionally, 167 differentially expressed genes (DEGs) were identified in the rutin vs. control groups, among which 52 were upregulated and 115 were downregulated. The relative expression of the DEGs among the samples is shown in a heatmap (Figure 2B, 2C).

### Gene ontology (GO) and Kyoto Encyclopedia of Genes and Genomes (KEGG) enrichment analyses of DEGs

**Figure 3:**
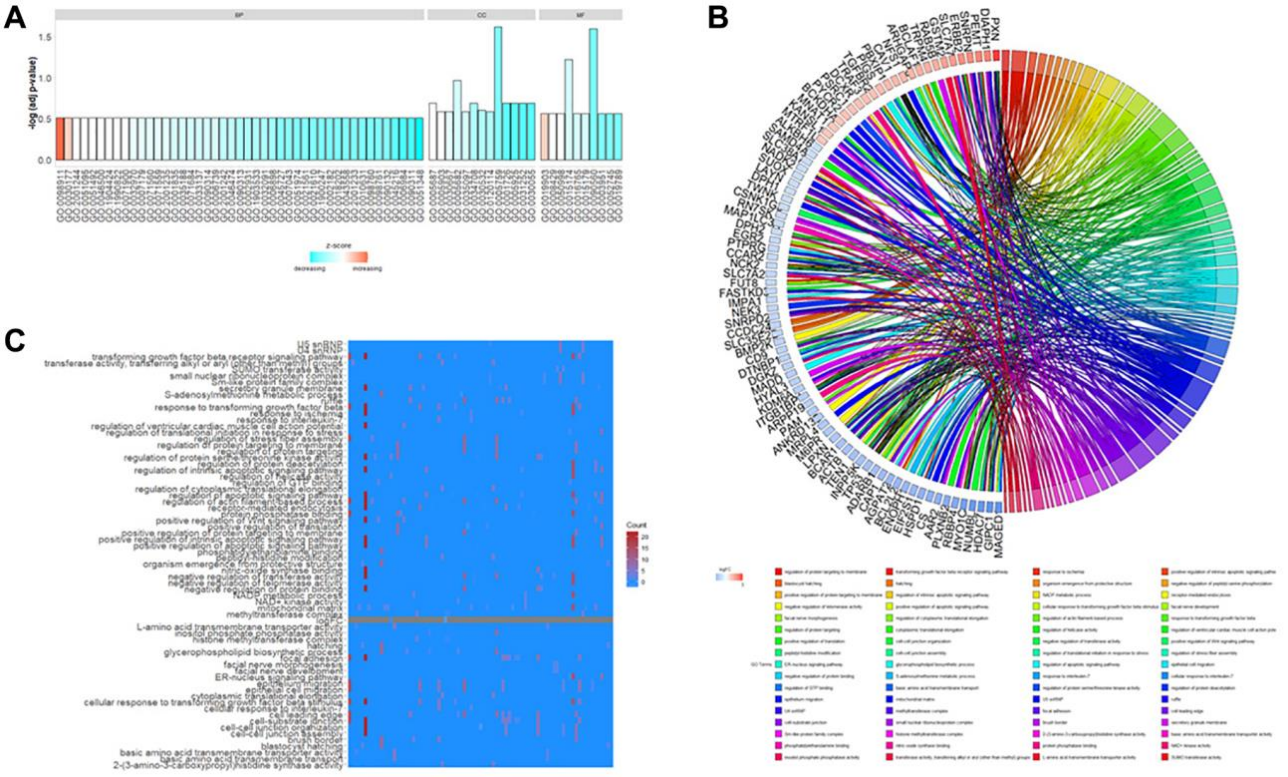
**Gene Ontology (G.O.) functional enrichment of differentially expressed genes (DEGs) in osteogenic differentiation of MSCs under the influence of rutin.** These genes were enriched in various biological processes (B.P.), cellular components (CC), and molecular function (M.F.) terms. (**A**) The main enrichment results of DEGs. The ordinate is indicated on a −log10 (*p*-value) scale. (**B**) Chordplot shows the G.O. enrichment results for key regulatory genes. The left semicircle represents the enriched key genes (the color of the squares refers to the relative amount of change logFC of the genes), and the right semicircle refers to the GO term enriched by the key genes in different colors and is connected by ribbons in the color representing the GO term. (**C**) Heatmap of G.O. enrichment results, where the vertical axis indicates G.O. terms. The horizontal axis is the enriched genes, and the square pigmentation indicates the number of GO terms enriched for a particular gene.

GO functional annotation of DEGs in rutin-treated MSCs during osteogenic differentation were enriched in various biological processes (BP), cellular components (CC), and molecular functions (MF.) terms (Figure 3A). In the BP category, the DEGs were mainly enriched in the regulation of ventricular cardiac muscle cell action potential (GO:0098911), positive regulation of the Wnt signaling pathway (GO:0030177), regulation of proteins targeting the membrane (GO:0090313), and negative regulation of transferase activity (GO:0051348). In the CC category, the DEGs were mainly enriched in the mitochondrial matrix (GO:0005759), U5 snRNP (GO:0005682), cell leading edge (GO:0031252), and cell-substrate junction (GO:0030055). In the MF category, the DEGs were mainly enriched in 2-(3-amino-3-carboxypropyl) histidine synthase activity (GO:0090560), basic amino acid transmembrane transporter activity (GO:0015174), protein phosphatase binding (GO:0019903), and SUMO transferase activity (GO:0019789). The relationship between the important DEGs enriched in GO analysis and the most enriched GO terms are indicated in Figure 3B. Additionally, the number of GO terms enriched by the DEGs are shown in Figure 3C.

**Figure 4:**
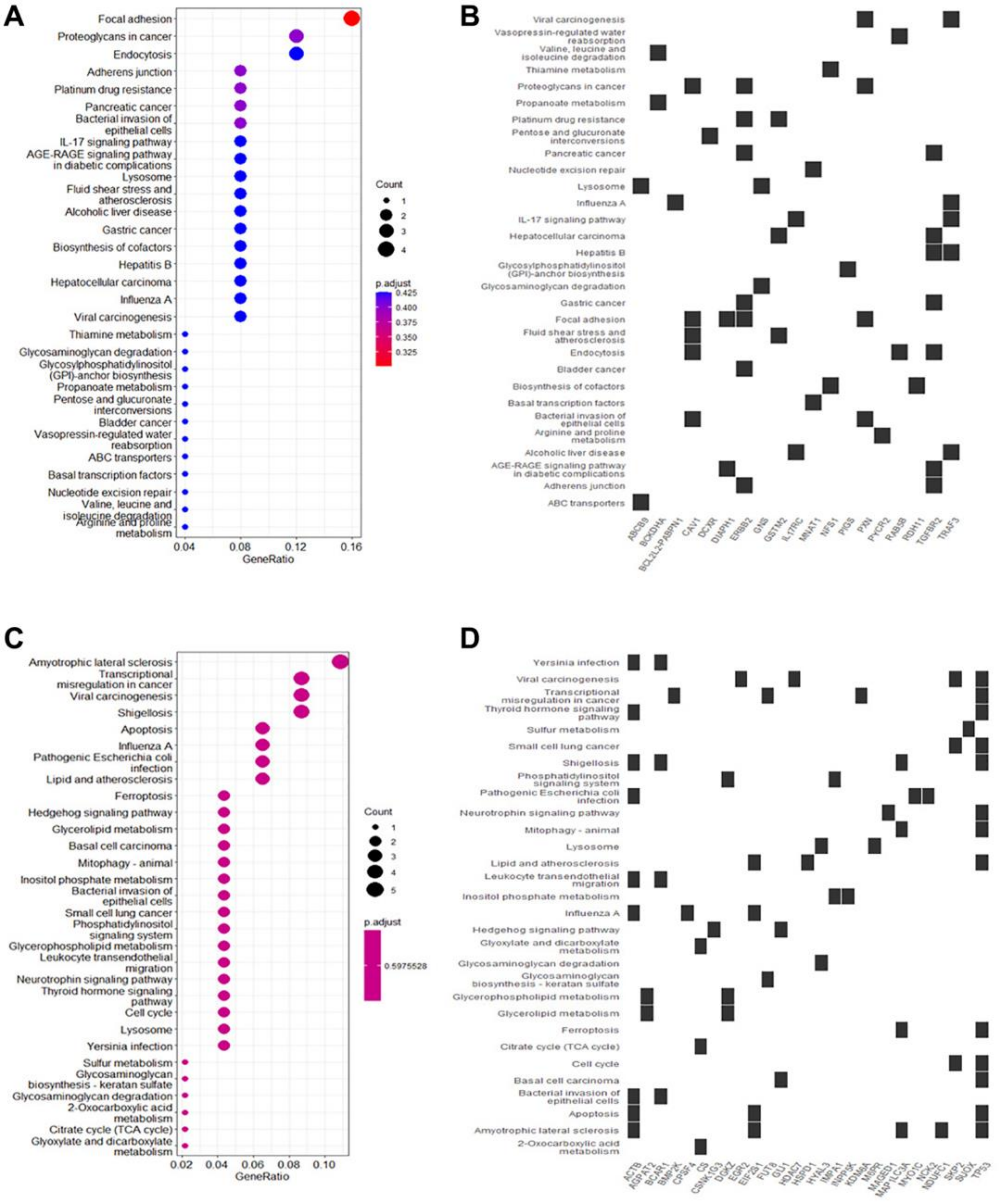
**Kyoto Encyclopedia of Genes and Genomes (KEGG) functional enrichment of DEGs in osteogenic differentiation of MSCs.** (**A**) The main enrichment results of the up-regulated genes (Count represents the number of enriched genes). (**B**) Heatmap of the relationship between up-regulated genes and KEGG terms. (**C**) The main enrichment results of the downregulated genes. (**D**) Heatmap of the relationship between downregulated genes and KEGG terms.

**Figure 5:**
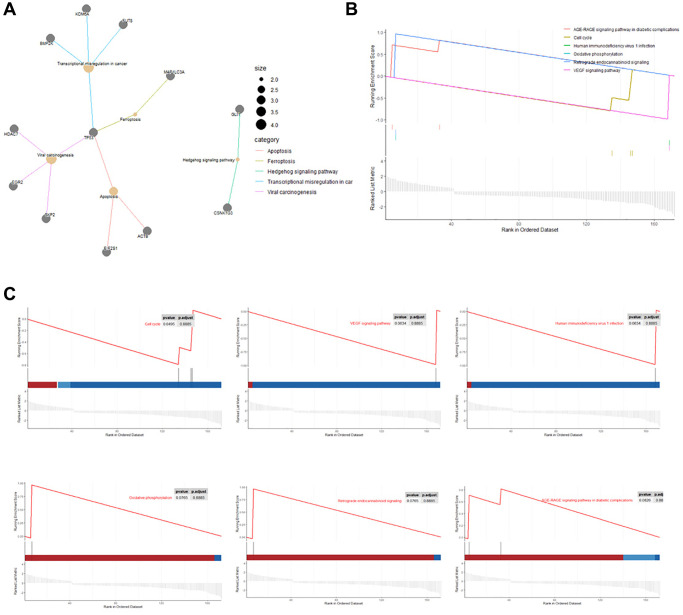
**KEGG network and Gene set enrichment analysis (GSEA).** (**A**) Cnetplot of the relationship between genes and KEGG terms (Size: Number of genes associated with KEGG pathway). (**B**, **C**) Gene set enrichment analysis results exhibited the top six enriched pathways.

KEGG pathway analysis showed that upregulated DEGs in rutin-treated MSCs during osteogenic differentation were mainly enriched in focal adhesion, adherens junction, IL-17 signaling pathway, and AGE-RAGE signaling pathway in diabetic complications (Figure 4A, 4B). Additionally, the downregulated DEGs were enriched in ferroptosis, transcriptional misregulation in cancer, Hedgehog signaling pathway, and apoptosis (Figure 4C, 4D). Further analysis of the enrichment data showed that TP53 is a key gene regulated by rutin, and is involved in the regulation of multiple pathways (Figure 5A).

Gene set enrichment analysis (GSEA) showed that the DEGs that were mainly enriched in cell cycle, VEGF signaling pathway, human immunodeficiency virus one infection, oxidative phosphorylation, retrograde endocannabinoid signaling, and AGE-RAGE signaling pathways in diabetic complications (Figure 5B, 5C).

### Rutin-targeted genes and core regulatory network

**Figure 6:**
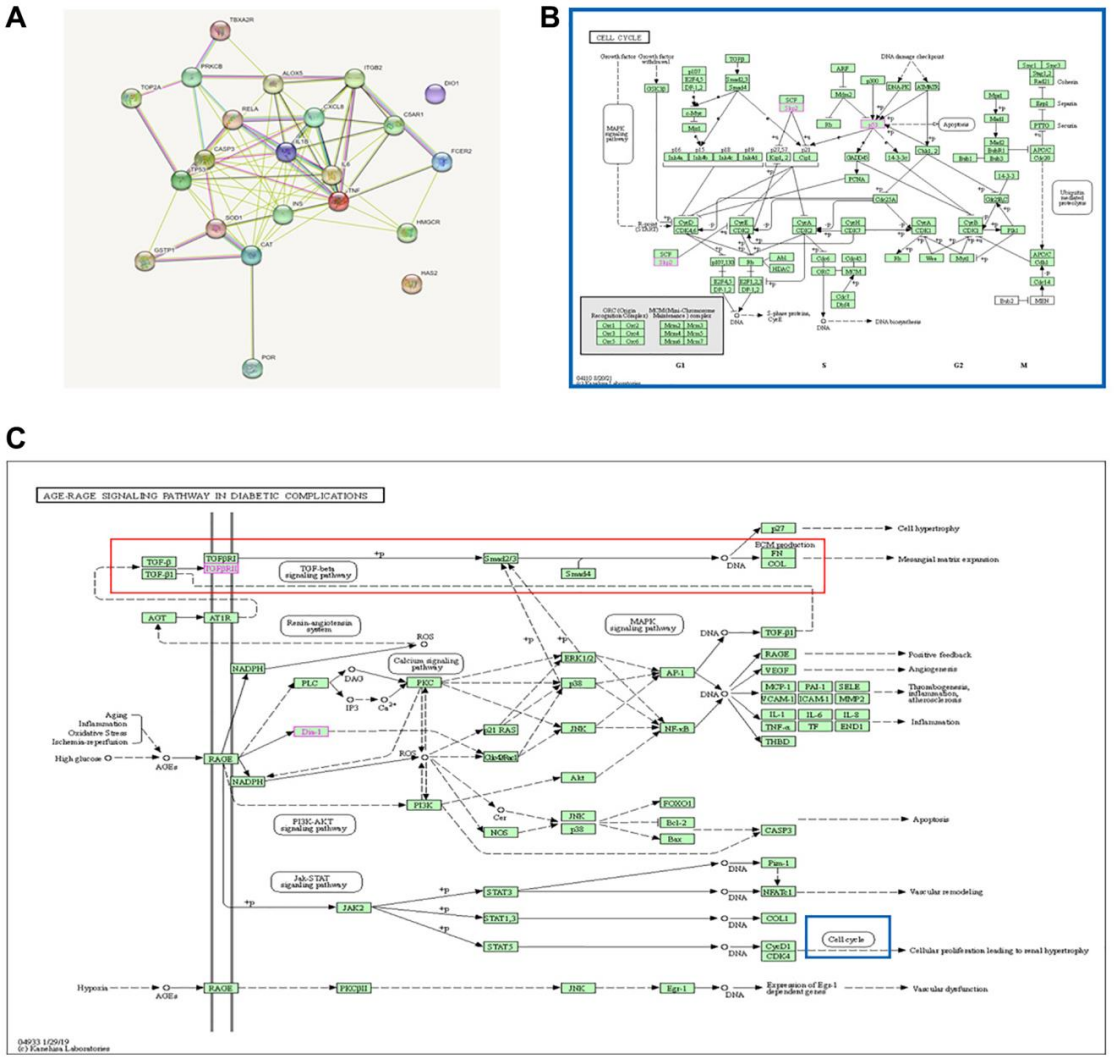
**Rutin-targeted genes and core regulatory network.** (**A**) PPI network of the 21 potential targets and the gene TP53. (**B**, **C**) Pathway diagram of enrichment results.

The keyword “Rutin” was searched on the Traditional Chinese Medicine Systems Pharmacology Database (TCMSP) (https://tcmsp-e.com/tcmsp.php) to identify potential targets of rutin. In total, 21 potential targets of rutin were identified in the TCSMP database. The 21 potential targets and the key gene TP53 were imported into the STRING database, resulting in 22 protein interactions. Notably, there was a strong association between TP53, CASP3 (caspase-3), and RELA (p65), which are the potential targets of rutin (Figure 6A). KEGG enrichment analysis showed that the 22 genes were mainly involved the AGE-RAGE signaling pathway in diabetic complication pathways. The AGE-RAGE signaling pathway in diabetic complication pathways mainly affects ECM deposition, and among them cell cycle regulation by TP53 is also involved (Figure 6B, 6C).

### Rutin inhibits TP53 (p53) activity and promotes ECM deposition during osteogenic induction

**Figure 7:**
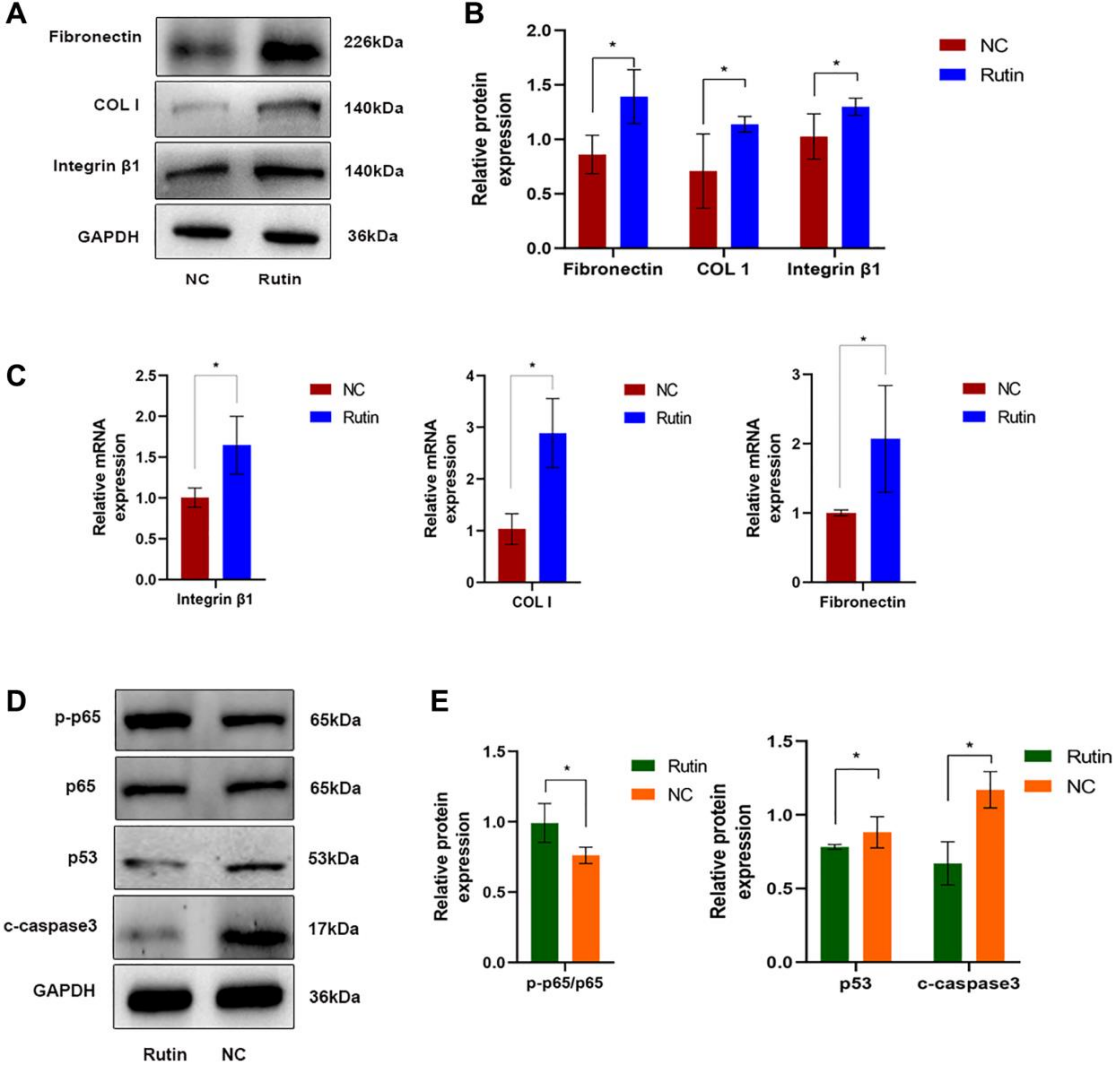
**Rutin inhibits TP53 (p53) activity and promotes ECM deposition during osteogenic induction.** (**A**, **B**) The protein expression of fibronectin, COL-I and integrin β1 in MSCs of the rutin and control groups. (**C**) Real-time RT-PCR analysis showed that the expression of ECM-related makers (fibronectin, COL-I) and integrin β1 were significantly enhanced by rutin treatment. (**D**, **E**) The protein expression of p65, p53, and cleaved caspase3 in MSCs of the rutin and control groups. The quantitative data were expressed as the mean ± S.D. of three independent experiments (^*^p < 0.05).

To further investigate the mechanisms through which rutin regulates osteogenic differentiation of MSCs, cells were treated with rutin, and the expression of ECM related genes were examined. Rutin treatment promoted ECM deposition (fibronectin and COL-I) and integrin β1 (affecting cell adhesion and migration) during osteogenic differentiation of MSCs (Figure 7A–7C). Additionally, western blotting showed that rutin treatment promoted p65 phosphorylation and inhibited p53 expression and caspase-3 cleavage (Figure 7D, 7E).

## DISCUSSION

Stem cells pluripotent, enabling them to develop into one of two distinct lineages: osteoblasts or adipocytes [17]. However, efficient differentiation of MSCs into osteoblasts is a significant obstacle in bone tissue engineering. Flavonoids are organic substances found in nature that possess numerous intrinsic properties and can affect both cell proliferation and bone metabolism [18]. Rutin, a widely occurring flavonoid, enhances periodontal tissues [19]. However, studies are yet to examine the effect of rutin on MSC differentiation.

In the present study, rutin treatment promoted osteogenesis in MSCs and induced the expression of osteogenesis-related genes. In contrast, rutin treatment inhibited the expression of adipogenesis- and differentiation-related genes in MSCs. Transcriptome and bioinformatic analyses showed that genes affected by rutin were primarily enriched in the AGE-RAGE signaling pathway in diabetic complications pathway, which regulates ECM deposition (Figure 6C red boxed section). ECM proteins determine cell shape and differentiation and participates in cell migration [20, 21]. Fibronectin promotes cell-matrix association [22]. Col-I and proteoglycans are basic skeletons that form a fibrillar meshwork complex on the cell surface [23]. Notably, most receptors are membrane integrins linked to cytoskeletal proteins. The ECM connects extracellular and intracellular regions through integrins, which facilitate the transmission of intracellular and extracellular signals [24]. Studies have shown that ECM promotes osteogenic differentiation of MSCs [25]. In the present study, rutin treatment significantly increased ECM deposition in MSCs, suggesting that rutin enhanced the osteogenic differentiation of MSCs by upregulating ECM deposition (Figure 7A–7C).

Further analysis identified p53 as a key regulatory gene involved in rutin-induced enhancement of osteogenic. Moreover, p53 is strongly associated with the predicted targets of rutin (p65 and caspase-3) in this study. Western blotting indicated that rutin treatment promoted p65 phosphorylation and inhibited the expression of p53 and cleaved caspase-3 (Figure 7D, 7E). p65 activation can inhibit p53 activity and caspase-3 cleavage, thereby promoting cell proliferation [26]. Rutin has been speculated to improve bone mass formation in patients with osteoporosis. MSCs differentiation is regulated by p53 through the control of genes related to the cell cycle and initial stages of differentiation. Loss of p53 in MSCs can induce alterations in bone remodeling via the negative regulation of osteoprotegerin [27]. Senescence regulation in MSCs is dependent on p53, and p53 downregulation can restore the osteogenic differentiation of MSCs [28].

MSC transplantation has a broad therapeutic spectrum and potential. For example, MSC transplantation is widely used for the treatment of various diseases, including graft-versus-host disease (GVHD), osteoarthritis, and asthma, as well as for the regeneration and repair of damaged tissues [29]. Additionally, MSCs possess considerable therapeutic potential for the treatment of cerebrovascular diseases [30]. Based on these findings, research on MSCs has shifted from basic to clinical applications. Rutin increases the proliferation and osteogenic differentiation of periodontal ligament stem cells (PDLSC) through GPR30-mediated PI3K/AKT/mTOR signal transduction [19] and indirectly promotes bone formation by increasing RANKL expression in osteoblasts [31]. However, the specific mechanism by which rutin promotes osteogenesis remains unclear. To the best of our knowledge, this study is the first to show that rutin promotes osteogenic differentiation of MSCs by decreasing p53 expression, increasing ECM deposition, and promoting p65 phosphorylation and caspase-3 cleavage.

Despite the promising findings, this study has some limitations. For example, this is an *in vitro* study with limited clinical translation. Therefore, it is necessary to investigate the efficacy and translational prospects of rutin-treated MSCs for bone regeneration using *in vivo* models. Additionally, other application prospects of rutin-treated MSCs are worth being investigated. Notably, rutin promotes the regeneration of periodontal tissues, indicating that it has the potential to be used to treat MSCs for dental bone grafting [32].

Conclusively, we demonstrated that rutin treatment improves osteogenic differentiation of MSCs. Our findings provide useful insights into the application of rutin in bone tissue engineering.

## MATERIALS AND METHODS

### Isolation and culturing of MSCs

Fresh umbilical cords were collected from a healthy 25-year-old female at the First Affiliated Hospital of China Medical University following cesarean delivery. The patients had no previous health condition prior to their inclusion in the study. Umbilical cords (6–8 cm) were processed in a Biosafety Cabinet-II (ESCO, Horsham, PA, USA) within 3 h of collection [33]. The cords were washed with PBS to eliminate blood clots and then sliced into 2–3 mm fragments to reveal Wharton's jelly. After 10–15 days, the MSCs attached themselves to the flask, displaying their characteristic morphological features. At 70–80% confluency, the cells were replaced by the addition of trypsin, which facilitated the separation of the adherent confluent cell monolayer. The floating cells were centrifuged at 11.18 rcf for 8 min, and the pellet was resuspended in media for cell subculture. Cells were maintained in DMEM supplemented with 10% FBS, 100 μg/mL of streptomycin, and 100 U/mL of penicillin at 37°C under a 5% CO_2_ atmosphere. The media were replaced every 2–3 days. Cell passages P2 and P3 were used for all the experiments.

### Osteoblast differentiation and analysis of MSCs

Briefly, approximately 500,000 human umbilical cord-derived MSCs (hUC-MSCs) were cultured in 6-well plates until 80% confluency was reached. The growth medium was replaced with osteoblast induction medium (OIM) for 24 h. The composition of OIM includes DMEM, 100 μG/mL of penicillin/streptomycin, 10% FBS, 10 mM of β-glycerophosphate, 2.5 μg/mL of ascorbic acid 2-phosphate, 0.1 μM of dexamethasone, and 2.5 μg/mL of amphotericin B. For subsequent experiments, a concentration of 20 μM was chosen for preconditioning of hUC-MSCs. The rutin group was exposed to 50 mg/mL of rutin (HY-N0148; MedChemExpress, Monmouth Junction, NJ, USA) for 24 h before experimentation.

### Lipogenic differentiation and analysis of MSCs

Briefly, approximately 500,000 MSCs were cultured in 6-well plates until 80% confluency was reached. The culture medium with a lipid differentiation medium containing 1 μM of dexamethasone, 10 μg/mL of insulin, and 500 μM of IBMX (Sigma Aldrich, Shanghai, China). For subsequent experiments, a concentration of 20 μM was chosen for preconditioning of hUC-MSCs.

### Real-time quantitative reverse transcription PCR (qRT-PCR)

Total RNA was extracted from the cells using the TRIzol reagent (Invitrogen, Carlsbad, CA, USA), followed by reverse transcription of RNA to generate cDNA using the MultiScribe Reverse Transcriptase Kit (Applied Biosystems, Waltham, MA, USA). qRT-PCR was performed on the PCR system using the SYBR Green kit (Bio Rad Laboratories Inc., Shanghai, China) and specific primers, according to the manufacturer’s instructions. GAPDH was used as the internal control. The primer sequence is listed below:

*alp* forwards 5′-GTGAACCGCAACTGGTACTC-3′; *alp* reverse 5′-GAGCTGCGTAGCGATGTCC-3′; *fabp4* forward 5′-AAGGTGAAGAGCATCATAACCCT-3′; *fabp4* reverse 5′-TCACGCCTTTCATAACACATTCC-3′; *pparg* forward 5′-TCCTGTAAAAGCCCGGAGTAT-3′; *pparg* reverse 5′-GCTCTGGTAGGGGCAGTGA-3′; *ocn* forward 5′-GGTGCAGACCTAGCAGACACCA-3′; *ocn* reverse 5′-AGGTAGCGCCGGAGTCTATTCA-3′; *runx2* forward 5′-GACTGGGGTTACCGTCATGGC-3′; *runx2* reverse 5′-ACTTGGTTTTTCATAACAGCGGA-3′; *opn* forward 5′-CTGCATACATGTAACCGCAGC-3′; *opn* reverse 5′-CTCTCCATAACATGGGC-3′; *fibronectin* forward 5′-GACCAGCAGAGGCATAAG-3′; *fibronectin* reverse 5′-CTCATCTCCAACGGCATAA-3′; *col-i* forward 5′-CCATTTATTAGTAGGTGTGCT-3′; *col-i* reverse 5′-TCACAAAAGAGTAGCCGAT-3′; *integrin β1* forward 5′-GCCTTACATTAGCACAACACC-3′; *integrin β1* reverse 5′-CATCTCCAGCAAAGTGAAAC-3′; *gapdh* forward 5′-TGGATTTTGGACGCATTGGTC-3′; *gapdh* reverse 5′-TTTGCACTGGTACGTGTTGAT-3′.

### Western blot analysis

Briefly, cells were lysed using a nuclear extraction kit (Novus Biologicals, Centennial, CO, USA), and the proteins were separated electrophoretically and transferred onto nitrocellulose membranes. Thereafter, the membranes were blocked with 5% milk at 37°C for 2 h, followed by incubation overnight with specific primary antibodies at 4°C. After washing, the membranes were incubated with specific secondary antibodies for 1 h at 37°C. Antibody reaction was detected via enhanced chemiluminescence (Yeasen, Shanghai, China). Antibodies used for western blot analysis are listed in Table 1.

**Table 1:** Antibodies used for western blot analysis.

**Target**	**Dilution ratios**	**Manufacturer**	**Item no.**
OCN	1:1000	Abcam, Cambridge, UK	ab93876
OPN	1:1000	Abcam, Cambridge, UK	ab214050
Runx2	1:1000	Abcam, Cambridge, UK	ab76956
Col I	1:1000	Abcam, Cambridge, UK	ab34710
Fibronectin	1:1000	Abcam, Cambridge, UK	ab2413
Integrin β1	1:1000	Abcam, Cambridge, UK	ab52971
p-P65	1:1000	Cell Signaling Technology, Boston, MA, USA	#78764
P65	1:1000	Cell Signaling Technology, Boston, MA, USA	#8242
P53	1:1000	Cell Signaling Technology, Boston, MA, USA	#2527
c-caspase3	1:1000	Abcam, Cambridge, UK	ab32351
GAPDH	1:5000	Abcam, Cambridge, UK	ab288151

### Alizarin red (AR) staining

AR staining was performed to detect mineralized calcium nodules. Briefly, MSCs were fixed with 4% paraformaldehyde for 10 min and stained with 1% AR solution (Shanghai Yuanye Biological, China) at 37°C for 30 min. After washing with PBS, stained cells and calcium nodules were observed under a microscope.

### MSC RNA-Seq and single-tubule RNA-Seq

MSC RNA-Seq and single-tubule RNA-Seq were performed as previously described [34]. Briefly, total RNA was extracted from MSCs using a Direct-Zol RNA MicroPrep kit (Zymo Research, Irvine, CA, USA), and reverse-transcribed to generate cDNA using a SMARTer V4 Ultra Low RNA kit (Takara Bio USA, Mountain View, CA, USA). cDNA fragments were targeted with barcodes using a Nextera XT DNA Sample Preparation Kit (Illumina, San Diego, CA, USA), followed by purification using AmPure XP magnetic beads (Beckman Coulter, Indianapolis, IN, USA) and quantified using a Qubit 2.0 Fluorometer (Thermo Fisher Scientific, Waltham, MA, USA). (Agilent Technologies, Wilmington, DE, USA). Libraries were pooled and sequenced on an Illumina HiSeq 3000 platform (Agilent Technologies, Wilmington, DE, USA) to yield an average depth of 60 million reads per sample.

### Data processing and transcript abundance quantification

Raw reads were processed using FastQC and aligned against the reference genome using STAR. Transcript abundance was quantified using RSEM5 and expressed as transcript per million (TPM).

### Bioinformatics analysis

Expression matrices were analyzed using intergroup and principal component analyses (PCA). Differential expression analysis was performed to identify differentially expressed genes (DEGs) between the rutin and control groups using the Bioconductor software package ‘limma’. Genes were considered differentially expressed at *p* < 0.05 and log2 fold change (FC) = mean ± 2SEM. Gene ontology (GO) functional annotation and Kyoto Encyclopedia of Genes and Genome (KEGG) pathway analysis of DEGs were performed to obtain crucial pathway information using the package ‘clusterProfiler’ in Bioconductor package. Enriched GO terms and KEGG pathways by the DEGs were visualized using R software. Additionally, DEGs were subjected to gene set enrichment analysis (GSEA) using the R package called 'fgsea,’ and the outcomes were visualized.

### Target prediction of rutin

To identify potential targets of rutin, the keyword “Rutin” was searched on the Traditional Chinese Medicine Systems Pharmacology Database (TCMSP) (https://tcmsp-e.com/tcmsp.php).

### Screening core targets

Rutin targets and core target TP53 were imported into the String database (https://string-db.org/) to generate a protein-protein interaction (PP1) diagram. The selected species for the analysis was human.

### Statistical analysis

All data are expressed as mean ± standard deviation (SD). Comparison between two groups was performed using Student’s *t*-test, with statistical significance set at *p* < 0.05. All statistical analyses were performed using SPSS 16.0 software.

### Data availability

The original data (western blot) presented in the study are included in the article/Supplementary Material; further inquiries can be directed to the corresponding authors.
